# Simulation of Drug Release from PLGA Particles *In Vivo*

**DOI:** 10.1155/2013/513950

**Published:** 2013-10-10

**Authors:** Kaori Sasaki, Martha Igarashi, Manami Hinata, Yuna Komori, Kouhei Fukushima

**Affiliations:** ^1^Laboratory of Gastrointestinal Tract Reconstruction, Tohoku University, Graduate School of Biomedical Engineering, Japan; ^2^Division of Surgical and Molecular Pathophysiology, Tohoku University, Graduate School of Medicine, 2-1 Seiryo-machi, Aoba-ku, Sendai 980-8575, Japan; ^3^Department of Colorectal Surgery, Tohoku University Hospital, 1-1 Seiryo-machi, Aoba-ku, Sendai 980-8574, Japan

## Abstract

Specific targeting of tissues and/or cells is essential for any type of drug delivery system because this determines the efficacy and side effects of the drug. Poly lactic-co-glycolic acids (PLGA) have long been used as biomaterials for drug delivery due to their excellent biocompatibility and biodegradability. Direct visualization of PLGA particles is feasible even within tissues, and cell specificity of the drug delivery system is normally assessed by using labeled particles. However, particle labeling alone does not address factors such as the release and distribution of the drug. Thus, it is desirable to set up a simulation system of drug release and distribution *in vivo*. In the present study, we aimed to establish a method to simulate drug distribution in PLGA drug delivery by using Hoechst 33342 as an imitating drug. Our approach enabled us to identify, isolate, and characterize cells exposed to Hoechst 33342 and to deduce the concentration of this fluorescent dye around both targeted and nontargeted cells. We believe that the method described herein will provide essential information regarding the specificity of cell targeting in any type of PLGA drug delivery system.

## 1. Introduction

Drug delivery systems (DDS) are designed to increase the therapeutic properties of a drug and reduce its side effects. Poly lactic-co-glycolic acids (PLGA), which have been approved by the US FDA, are frequently used as biomaterials for drug delivery due to their excellent biocompatibility and biodegradability [1]. PLGA particles are prepared by single- or double emulsion-solvent evaporation. In particular, a water-in-oil-in-water (w/o/w) method is widely used to encapsulate water soluble drugs [2]. The mechanism of degradation of PLGA particles generally involves a hydrolytic process.

The maximum effect of a drug can only be achieved by strictly controlling target cell specificity. Moreover, reduced exposure of nontargeted cells to the drug may prevent undesirable side effects. In the context of *in vivo* distribution of PLGA “particles,” visualization of the particles themselves is feasible when markers such as fluorescent dyes are used [3–5]. However, other details of the DDS, such as the type and number of cells exposed to the drug, *in situ* drug concentration, and functional consequence for each cell population after drug exposure, are often more difficult to determine. These problems frequently arise with PLGA DDS. For example, although drug behavior depends on the chemical properties of the drug in question, the distribution of the drug is also affected by other factors. The nature of individual PLGA particles as a carrier varies depending on the monomer ratio, particle size/size distribution, morphology, and the presence/absence of additives [1], all of which determine the rate of degradation of the particles. The route and method of administration and microenvironment at the targeted site are also relevant factors that need to be considered. The microenvironment of target tissues is composed of various types of cells, extracellular matrix, and flow of extracellular fluid determined by tissue dynamics, all of which are variable in an individual target tissue or organ. Thus, there is a need to develop a system that can be used to assess the distribution of drugs incorporated into PLGA particles. Fluorescence can be used to visualize labeled proteins (e.g., GFP-fusion proteins) and/or genes in order to analyze their release into the tissue microenvironment. However, this approach using labeled materials is not always straightforward. For example, constructs must be developed and the detection limit is usually quite low unless there is aggregation of the fluorescent materials to specific cellular components. The types of factors that need to be monitored include (i) time-dependent release of drugs, (ii) the drug concentration to which targeted and nontargeted cells are exposed, (iii) the types and character of cells exposed to the drug, and (iv) functional changes to the cells after drug exposure. These factors vary for individual PLGA particles depending on the method of administration and the type of targeted tissue.

Hoechst 33342 (2′-[4-ethoxyphenyl]-5-[4-methyl-1-piperazinyl]-2,5′-bi-1H-benzimidazole trihydrochloride trihydrate) is a fluorescent dye, that is, excited by ultraviolet light at 361 nm, and emits blue/cyan fluorescent light with an emission maximum at about 486 nm. Fluorescence is enhanced upon binding to double-stranded DNA. Because of this enhancement in fluorescence, Hoechst 33342 is used for the quantification of DNA and particularly for staining the nuclei of living and fixed cells. This dye is also used as a powerful tool in the purification and characterization of stem cells of variable lineages [6, 7].

In the present study, we intended to establish a method to simulate drug distribution in PLGA drug delivery *in vivo* using Hoechst 33342 as an imitating drug. The present approach enables us to identify, isolate, and characterize specific cells exposed to Hoechst 33342 and to infer the likely concentration of this fluorescent dye in the microenvironment around the particles.

## 2. Materials and Methods

### 2.1. Reagents and Media Used in This Study

We obtained Dulbecco's Modified Eagle Medium (D-MEM), RPMI 1640, 0.25% (w/v) trypsin and 1 mM ethylenediaminetetraacetic acid (EDTA), verapamil hydroxyl chloride, PLGA, methylene chloride, and polyvinyl alcohol from Wako Pure Chemicals Ltd. (Osaka, Japan). Fetal calf serum (FCS) was from Sanko Junyaku Co., Ltd. (Tokyo, Japan). Antibiotics (penicillin and streptomycin) and the MTT assay kit were from Nacalai Tesque Co. (Kyoto, Japan). Hoechst 33324 was purchased from Sigma-Aldrich Japan K.K. (Tokyo, Japan), and 3,3′-dioctadecyloxacarbocyanine perchlorate (Dio) and Cell Mask Plasma Membrane Stain were from Invitrogen Japan K.K. (Tokyo, Japan). Optimal cutting temperature (OCT) compound was from Sakura Co. (Tokyo, Japan).

### 2.2. Cellular Toxicity of Hoechst 33342 and PLGA Particles

IEC-6 cells (a rat small intestinal epithelial cell line) and U-937 cells (a human myeloid cell line) were provided by the RIKEN BRC through the National Bio-resource Project of the MEXT, Japan. IEC-6 cells are nontransformed crypt-like cells isolated from the whole small intestine [8]. The U937 cell line is a human cell line established from the pleural effusion of a patient with diffuse histiocytic lymphoma and displaying many monocytic characteristics [9]. IEC-6 cells were routinely grown in D-MEM containing 5% FCS and 0.1% antibiotics (Penicillin and Streptomycin) at 37°C in a 5% CO_2_ atmosphere. U-937 cells were similarly grown in RPMI1640 containing 10% FCS and 0.1% antibiotics.

IEC-6 or U-937 cells were grown on 96-well plates for 2 days. Serial amounts of Hoechst 33342 (ranging from 0 to 5 *μ*g/mL) or PLGA particles incorporated with phosphate-buffered saline (PBS) only (ranging from 0 to 250 *μ*g/mL) were then added to the medium and the cell culture was continued. The cells were viewed and photographed under phase contrast microscopy (CKX31; OLYMPUS Co., Tokyo, Japan) after 1, 2 or 4 days. A cell viability assay was also carried out using the MTT assay kit according to the manufacturer's protocol. The absorbance at 570 nm was determined using a microplate reader, FLEX station 3 (Molecular Device Japan Co., Tokyo, Japan). The data were represented as the mean of triplicate determinations normalized to the control value, which was arbitrarily set at 100%.

### 2.3. Relationship between Concentration of Hoechst 33342 and Fluorescence Intensity

To determine whether the concentration of Hoechst 33342 correlates with fluorescence intensity of stained cells, IEC-6 cells were grown on 96 well plates. The medium was exchanged once the cells had reached confluency. The cells were then exposed to serial amounts of Hoechst 33342 (ranging from 0 to 1 *μ*g/mL) for a period of 24 hrs. These experiments were performed in quadruplicate. Fluorescence intensity of each well was measured using a FXEX station 3 scanning fluorometer with an excitation at 355 nm and emission at 460 nm. After measurement the medium of each well was removed. The cells were then washed with PBS and incubated with 20 *μ*L of 0.25% (w/v) trypsin and 1 mM EDTA for 5 minutes to detach the cells from the plate. The number of cells from each well was counted after staining with 0.25% Trypan Blue, and the values were expressed as fluorescent intensity/1000 cells. The experiment was also conducted using U-937 cells essentially as described above, except in this case that the trypsin-EDTA treatment step was omitted. IEC-6 and U-937 cells were also analyzed using the flow cytometer, FACS Aria II (BD Biosciences Japan, Tokyo, Japan).

Certain types of cells, such as hematopoietic and epithelial stem cells, are able to efflux Hoechst 33342 through the MDR-1-encoded triphosphate-binding cassette (ABC) transporter [10]. In such cases, fluorescence intensity of the cells may decrease due to efflux of the dye. Therefore, we examined the requirement of verapamil, a blocker of the efflux of a variety of DNA-binding fluorochromes, including Hoechst 33342, in the measurement of fluorescence intensity. To do this we set up additional cultures using IEC-6 cells in the presence of a serial amount of Hoechst 33342 and 50 *μ*M verapamil hydroxyl chloride.

Frozen tissue sections are usually prepared to allow histological investigation. However, fluorescence intensity of cells stained with Hoechst 33342 *in vivo *may be affected by the preparation of the frozen tissue sections. Therefore, we compared the fluorescent intensity of IEC-6 cells stained with Hoechst 33342 before and after treatment (i.e., fixation, dehydration, and freezing). IEC-6 cells were cultured on a 96 well plate and incubated with 100 ng/mL Hoechst 33342 for 24 hrs in quadruplicate. The cells were then washed with PBS and their fluorescence intensity was measured. Next, the cells were fixed with 4% paraformaldehyde for 1 hr, dehydrated with 5, 10, and 15% sucrose in PBS, and frozen at −80°C for 1 hr. Fluorescence intensity was then remeasured and the cell number of each well was counted. Finally, values of fluorescent intensity/1000 cells were calculated.

### 2.4. Preparation of Hoechst 33342-Incorporated PLGA Particles

Hoechst 33342-incorporated PLGA particles were prepared according to the oil/water emulsion/solvent evaporation method described by Tsung et al. with some minor modifications [11]. In brief, 20 *μ*L of 1 mg/mL Hoechst 33342 was added to 500 *μ*L of methylene chloride containing 25 mg of PLGA (lactic acid: glycolic acid = 75 : 25). In some experiments, the particles were also labeled with Dio, a lipophilic tracer, by the addition of Dio into methylene chloride at a concentration of 0.01% (w/v) (4). The mixture of Hoechst 33342 and methylene chloride was stirred thoroughly using a homogenizer (HG-200; HSIANGTAI Machinery Industry Co., Ltd. Taipei, Taiwan) at 12000 rpm for 15 seconds. Then, 5 mL of 1% wt/vol polyvinyl alcohol was combined with the solution above and emulsified using a sonicator (Vibra Cell; SONIC & MATERIALS Inc., Newtown, CT USA) set to 40% power for 20 seconds. Finally, the resulting emulsion was stirred overnight to evaporate the methylene chloride. The particles were then collected and washed with PBS and analyzed with a Zeta-Potential & Particle Size Analyzer ELSZ-2 (Otsuka Electronics, Osaka, Japan). Next, we calculated the incorporation rate of Hoechst 33342. A 1 mL aliquot of emulsion was centrifuged at 15000 g for 10 min before washing with PBS. The supernatant was then collected and the concentration of Hoechst 33342 was measured. The incorporation ratio of Hoechst 33342 was calculated using the value of Hoechst 33342 concentration and the amount of supernatant. Unloaded PLGA particles were also synthesized to study cellular toxicity of PLGA particles alone.

### 2.5. *In Vitro* Release of Hoechst 33342

When *in vitro* release of Hoechst 33342 from the particles was investigated, 3 mL of Hoechst 33342-incorporated PLGA particles were combined with 7 mL saline and incubated at 37°C in a shaking bath. A small amount of incubation solution was collected after 0, 1, 2, 3, and 4 days, and the concentration of Hoechst 33342 in each sample was determined. To quantify the concentration of dye we combined 180 *μ*L of either sample or a solution containing serial amounts of Hoechst 33342 (ranging from 0 to 1000 *μ*g/mL) as a control with 20 *μ*L a solution containing 20 ng of mouse genomic DNA in a 96-well plate format. The fluorescent intensity of each well was then measured using a FXEX station 3. This experiment was performed in duplicate and mean values of fluorescent intensity were calculated.

### 2.6. *In Vivo* Experiments Using Hoechst 33342-Incorporated PLGA Particles in the Absence or Presence of Dio-Labeling

This project was approved by the Ethics Committee for the Care and Use of Laboratory animals of Tohoku University School of Medicine. C57/BL6 mice (8 to 12 weeks old) were housed in the animal room at Tohoku University Institute for Experimental Animals, Sendai, Japan, with a 12-hour light/dark cycle. The mice were fed a standard murine diet and allowed tap water *ad libitum*. Hoechst 33342-incorporated PLGA particles dissolved in 200 *μ*L PBS were administered to the mice using one of three routes: (i) intravenous administration *via* the caudal vein, (ii) local injection into the femoral muscle, or (iii) intraperitoneal injection. Mice were sacrificed by cervical dislocation and organs or tissues of interest were removed, fixed with 4% paraformaldehyde, dehydrated in 10, 15, and 20% sucrose PBS, mounted in OCT compound, and frozen and stored at −80°C until required. Frozen sections of 5 *μ*m in thickness were prepared, washed in PBS, mounted in the water soluble mounting medium, and observed by fluorescence microscopy (model BZ-8100 microscope; KEYENCE, Tokyo, Japan) with or without staining of the plasma membrane with CellMask Plasma Membrane Stain. 

When the particles without Dio-labeling were administered into the peritoneal cavity, intraperitoneal macrophages were collected 20 or 60 hours after administration. In brief, ice cold PBS was poured into the abdominal cavity, recovered, and centrifuged for 10 minutes at 135 g. Number, viability, and purity of the cells were evaluated by Trypan Blue exclusion. The isolated cells were analyzed using FACS Aria II, and the fluorescence intensity was compared with that of U-937 cells incubated with 0, 10, 100, or 1000 ng/mL Hoechst 33342 as a control. Fluorescence intensity of peritoneal macrophages and U-937 cells was measured as described above using a FXEX station 3 scanning fluorometer, and values were expressed as fluorescent intensity/10000 cells. Next, we estimated the concentration of Hoechst 33342 to which the peritoneal macrophages had been exposed based on the control experiment.

## 3. Results and Discussion

The initial pharmacokinetic study in DDS using PLGA was to investigate the tissue distribution of PLGA particles, which can be visualized by labeling with a fluorescent dye [3]. However, the essential aim of this investigation was not only to determine the localization of particles but also to analyze the kinetics of drug release and efficacy of cell targeting. 

**Figure 1:**
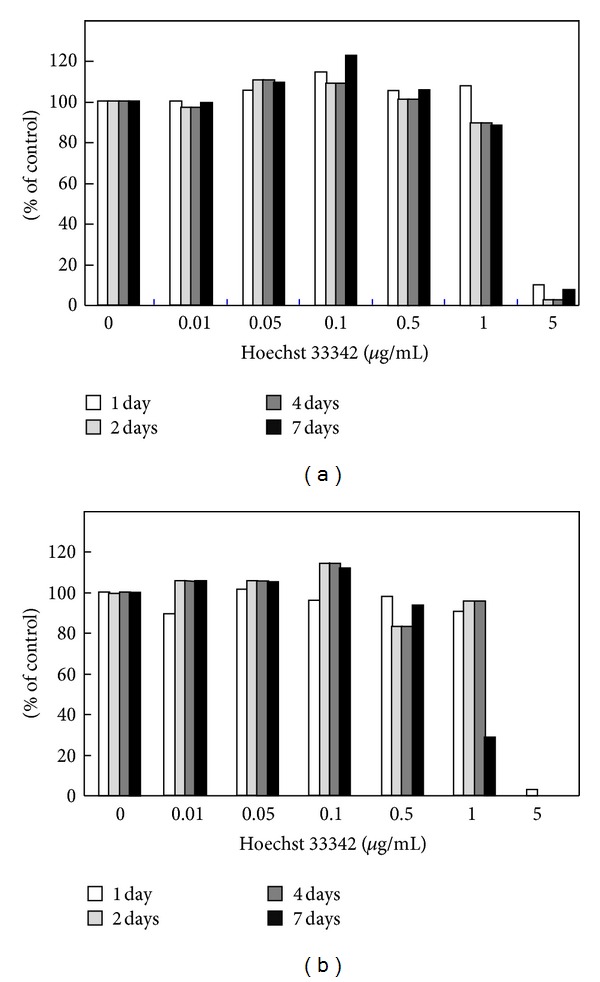
Effect of Hoechst 33342 concentration on the viability of IEC-6 (a) and U-937 cells (b). Both cell types were treated with different concentrations of Hoechst 33342 (0 to 5 *μ*g/mL) for up to 7 days. Cell viability was then determined by using the MTT assay. The data are expressed in terms of the percentage of viable cells relative to control cells, which were treated with medium only.

**Figure 2:**
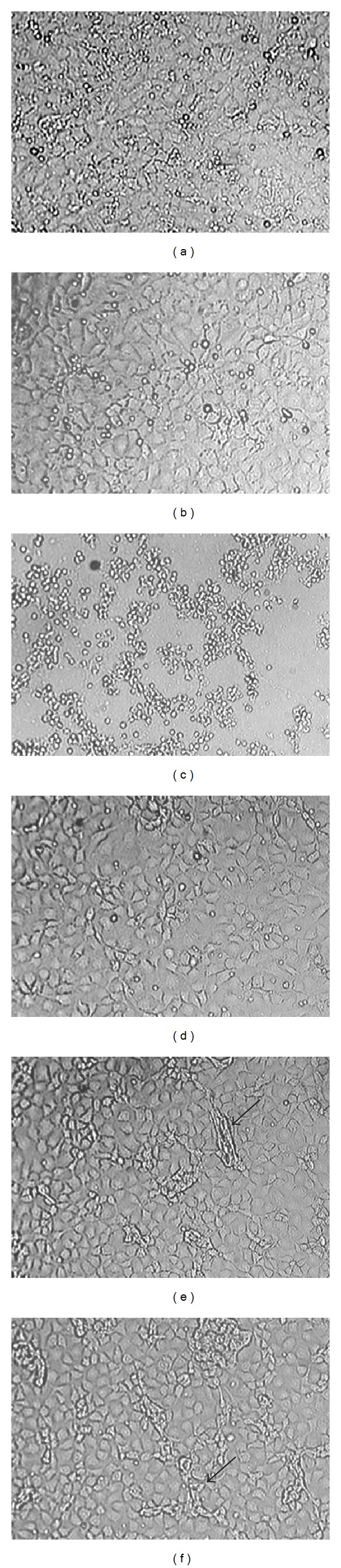
Phase contrast microscopy images of IEC-6 cells cultured with Hoechst 33342. (a), (b), and (c) show cultures grown in the absence of Hoechst 33342 (a), or in the presence of 1 (b), or 5 *μ*g/mL Hoechst 33342 (c) for 1 day. Note that many cells were detached when 5 *μ*g/mL of dye was used. (d), (e), and (f) show cultures grown in the presence of 1 *μ*g/mL Hoechst 33342 for 4 (d), 7 (e), or 12 days (f). Note that bundle-like structures (indicated by *arrows*) were observed in (e) and (f).

**Figure 3:**
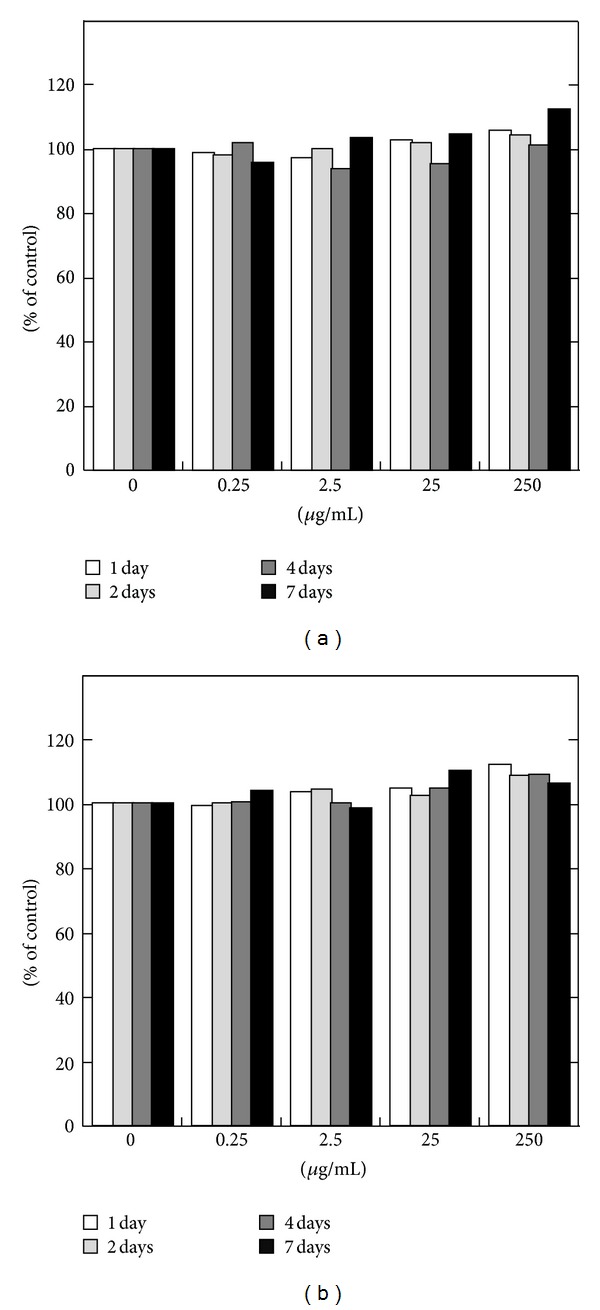
Effect of PLGA particles on the viability of IEC-6 (a) and U-937 cells (b). PLGA particles were incorporated with PBS. Both cell types were treated with different concentrations of PLGA particles (0 to 250 *μ*g/mL) for up to 7 days. Cell viability was then determined by using the MTT assay. The data are expressed in terms of the percentage of viable cells relative to control cells, which were treated with medium only.

In the present study we used Hoechst 33342 as an imitating drug and initially examined the effects of Hoechst 33342 on cell viability. MTT assays demonstrated that Hoechst 33342 appeared to be nontoxic up to a concentration of 1 *μ*g/mL in two different cell types, epithelial and myeloid cells, at least within 4 days of exposure (Figures 1 and 2). Hoechst 33342 was found to be highly toxic and induced cell death at a concentration of 5 *μ*g/mL (Figure 2(c)). When IEC-6 cells were cultured with 1 *μ*g/mL Hoechst 33342 for 7 or 12 days, bundle-like structures were detected, suggesting that long-term culture in the presence of high concentrations of Hoechst 33342 may affect epithelial phenotype (Figures 2(e) and 2(f)). PLGA particles themselves were also nontoxic as shown in Figure 3.

**Figure 4:**
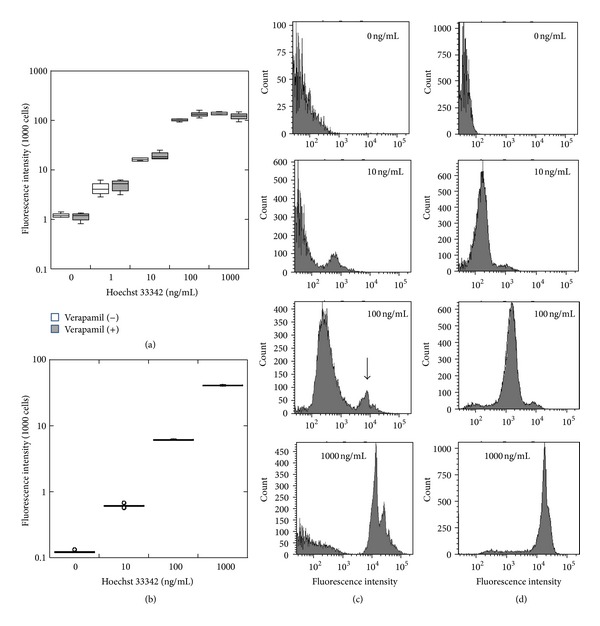
(a) Dose response relationship between Hoechst 33342 and fluorescence intensity in the presence or absence of 50 *μ*M verapamil in IEC-6 cells, (b) Hoechst 33342 dose response for fluorescence intensity in IU-937 cells, and (c) FACS analysis of IEC-6 cells incubated with 0, 10, 100, or 1000 ng/mL Hoechst 33342. Note that the small peak (indicated by an arrow) is also observed at 100 ng/mL of dye. (d) FACS analysis of U-937 cells incubated with 0, 10, 100, or 1000 ng/mL Hoechst 33342.

In the next step we measured fluorescence intensity of cells incubated in the presence of serial amounts of Hoechst 33352. Fluorescence intensity was clearly dose-dependent in both IEC and U-937 cells (Figures 4(a) and 4(b)). When we compared fluorescent intensity between IEC-6 and U-937 cells exposed to the same concentration of dye, IEC-6 cells exhibited a greater fluorescence. These observations suggest that fluorescence intensity depends, at least in part, on cell type, that is, possibly related to nuclear size as well as other factors [10]. We also examined whether 50 *μ*M verapamil, which blocks ABC transporters, decreased the fluorescence intensity. However, verapamil had only a minimal effect on the fluorescence intensity of IEC-6 cells (Figure 4(a)). The flow cytometric analysis also demonstrated that fluorescence intensity was dose dependent of Hoechst 33342. Interestingly two peaks were observed in IEC6 cells incubated with 100 ng/mL Hoechst 33342, suggesting that fluorescent intensity may not be uniform even in the same type of cells, probably due to the heterogeneity of the IEC-6 cells in the cell cycle.

**Figure 5:**
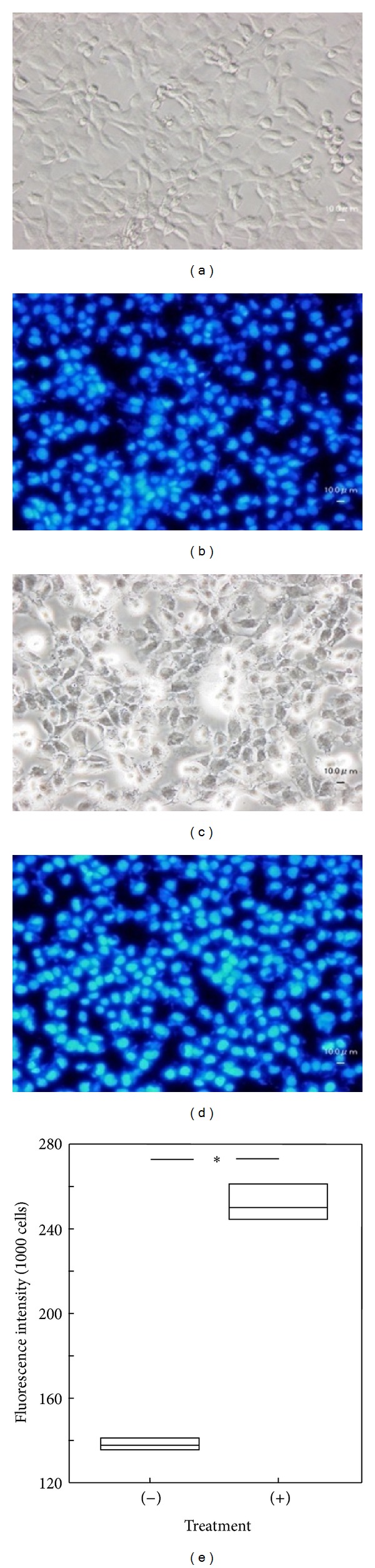
Effect of fixation, dehydration, and freezing of Hoechst 33342-stained IEC-6 cells on fluorescence intensity. IEC-6 cells stained with 100 ng/mL Hoechst 33342 were observed by both phase contrast and fluorescent microscopy before ((a), (b)) and after treatment ((c), (d)). Fluorescence intensity was also measured before and after treatment (e). Significant differences were detected as shown by an asterisk (*P* < 0.05).

We also investigated whether the way in which frozen tissue sections were prepared might have an effect on the fluorescent intensity of the cells. To simulate the preparation of frozen tissue sections we fixed, dehydrated and froze Hoechst 33342-stained IEC-6 cells, and then compared the fluorescence intensity before and after treatment. However, this treatment resulted in only a slight increase, rather than decrease, in fluorescence intensity (Figure 5).

**Figure 6:**
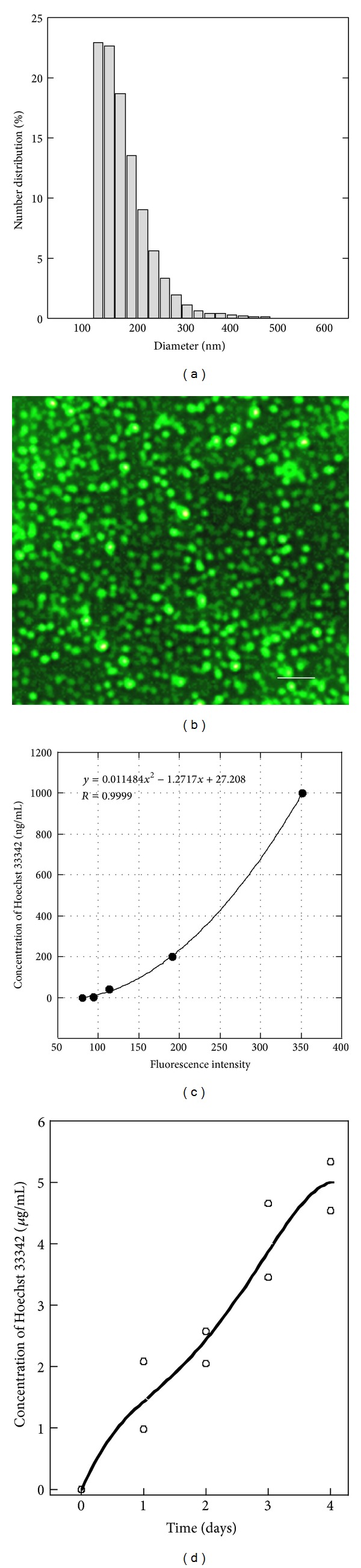
Distribution in the diameter of Dio-labeled and Hoechst 33342-incorporated PLGA particles. (a) The particles were pictured under fluorescent microscopy. (b) The size bar represents 5 *μ*m. (c) Standard curve for measuring Hoechst 33342 concentration. (d) *In vitro* release of Hoechst 33342 from the Dio-labeled and Hoechst 33342-incorporated PLGA particles.

In the next step we prepared Dio-labeled and Hoechst 33342-incorporated PLGA particles. The mean particle size and zeta potential were 333.8 nm and −2.14 mV, respectively (Figures 6(a) and 6(b)). The concentration of Hoechst 33342 in the supernatant of PLGA emulsion was 2.8 *μ*g/mL, suggesting that 14 *μ*g of Hoechst 33342 was contained in the aqueous phase. Because we used 20 *μ*g of Hoechst 33342 in total, the % entrapment of Hoechst 33342 was calculated as 30%. We observed the time-dependent increase of Hoechst 33342 concentration in the *in vitro* release experiment (Figures 6(c) and 6(d)).

**Figure 7:**
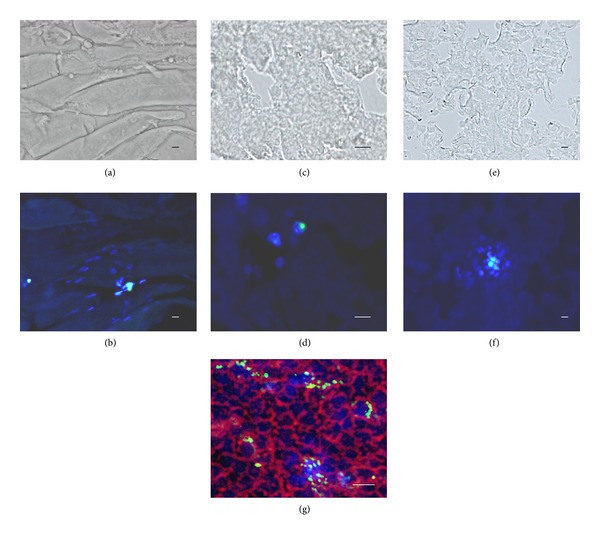
Frozen tissue sections of the femoral muscle, liver, lung, and spleen. The Dio-labeled and Hoechst 33342-incorporated PLGA particles were locally injected into the femoral muscle or introduced intravenously through the caudal vein. The femoral muscle ((a), (b)), liver ((c), (d)), lung ((e), (f)), and spleen (g) were removed 3 days after administration of the particles. Cryostat sections were observed by phase contrast ((a), (c), and (e)) or fluorescent microscopy ((b), (d), (f), and (g)). Green, blue, and orange colors represent particles labeled with Dio ((b), (d), (f), and (g)), nuclear staining with Hoechst 33342 ((b), (d), (f), and (g)), or plasma membrane stained with CellMask Plasma Membrane Stain (g), respectively. Bars indicate 10 *μ*m. Note that nuclear staining is only observed around the particles.

Particles were administered to the mice by one of three different methods: (i) direct injection into the femoral muscle, (ii) intravenous administration, or (iii) intraperitoneal injection. Frozen tissue sections from the femoral muscle revealed nuclear staining with blue fluorescence around the green particles and lack of nuclear staining in the muscle away from the particles (Figures 7(a) and 7(b)). When we administered the particles through the caudal vein to mice, the particles were trapped in the liver, lung, and spleen. For any tissue examined, nuclear staining was only detected in cells in close proximity to the particles and not in cells separate from the particles (Figures 7(c), 7(d), 7(e), 7(f), and 7(g)). We used an additional fluorescent dye, Dio, to label the PLGA particles themselves. Dio-labeling facilitated the detection of the particles in tissue sections. Although the emission spectra of Hoechst 33342 and Dio partly overlap, the pattern of nuclear staining appears to be minimally affected because of the differential emission peak wavelength (461 nm for Hoechst 33342; 501 nm for Dio) and their respective affinities to distinct cellular components (Hoechst 33342, high affinity for nuclear DNA; Dio, high affinity for the plasma membrane). In practice, we did not observe any nuclear staining *in situ* when the Dio-labeled particles without Hoechst 33342-incorporation were used (data not shown).

**Figure 8:**
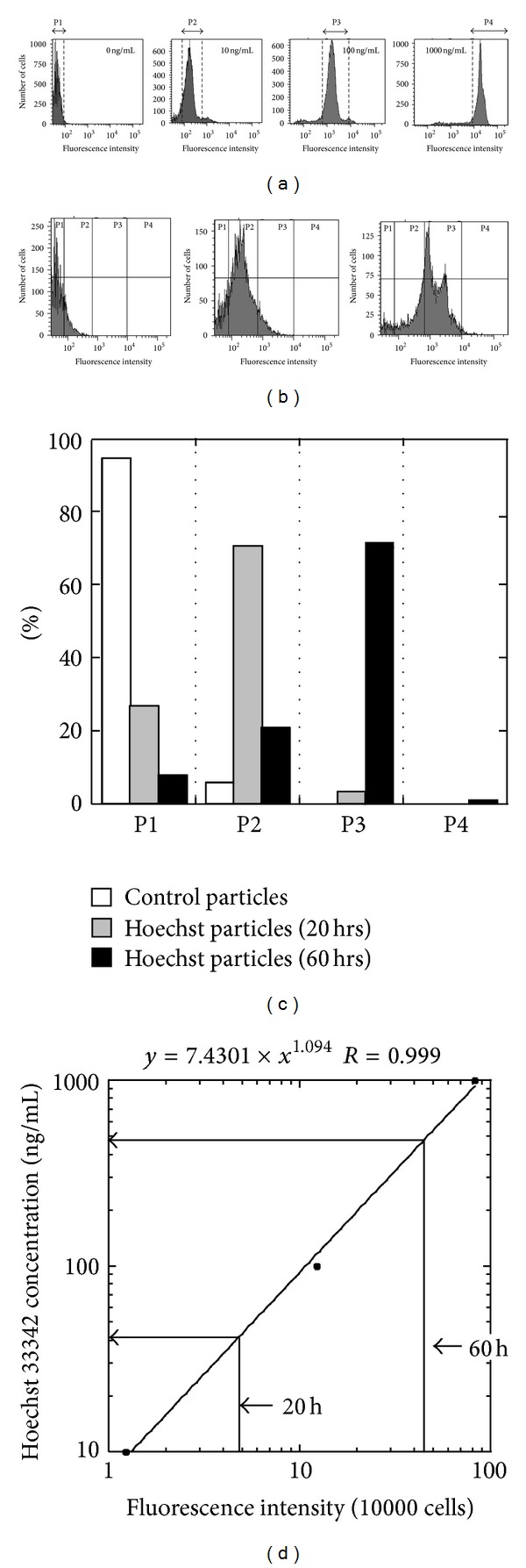
(a) Fluorescence intensity of U-937 cells was analyzed after staining with serial concentrations of Hoechst 33342 using FACS Aria II. The segments P1, P2, P3, or P4 correspond to the range of fluorescence intensity at 0, 10, 100, or 1000 ng/mL Hoechst 33342, respectively. (b) FACS analysis of isolated peritoneal macrophages from mice injected with the control (*left*) or Hoechst 33342-incorporated particles (*middle* and *right*). The cells were isolated 20 (*middle*) or 60 hours (*right* and *left*) after injection. P1, P2, P3, and P4 indicate the above range of fluorescence intensity at 0, 10, 100 or 1000 ng/mL Hoechst 33342, respectively. (c) The ratio of isolated peritoneal macrophages with fluorescence intensity that fell into segments P1, P2, P3 or P4. (d) Estimated mean concentration of Hoechst 33342 to which the peritoneal macrophages were exposed. The standard curve was generated using U-937 cells exposed to 10, 100, or 1000 ng/mL Hoechst 33342.

Finally, we simulated characterization of cells isolated from mice after administration of Hoechst 33342-incorporated PLGA particles. We hypothesized that the particles gradually released Hoechst 33342 after peritoneal injection, resulting in a time-dependent increase in the concentration of Hoechst 33342 and enhancement of nuclear staining intensity of peritoneal macrophages in the peritoneal cavity. To test this hypothesis we isolated macrophages from the peritoneal cavity of mice injected with the control and Hoechst 33342-incorporated particles and then compared their staining pattern to that of U-937 cells incubated with serial amounts of Hoechst 33342. We divided the range of fluorescence intensity into the four segments. We defined P1, P2, P3, and P4 segments as the range corresponding to the fluorescent intensity of U-937 cells incubated with 0, 10, 100, or 1000 ng/mL Hoechst 33342, respectively (Figure 8(a)). The cells from mice receiving the control particles showed similar cell distribution to that of U-937 cells without Hoechst 33342 (Figure 8(b)). Over 90% of the cells were included in the P1 segment (Figure 8(c)). When we examined the cells 20 hrs after the injection of the Hoechst 33342-incorporated particles, the peak in cell number shifted to the right and a large population of the cells (70%) fell into the P2 segment. We next examined the cells isolated 60 hrs after injection. Two peaks were observed in the P3 segment with the majority of cells (70%) falling into this segment (Figures 8(b) and 8(c)). From the data we calculated the mean Hoechst 33342 concentration to which the isolated cells had been exposed in the peritoneal cavity. We constructed the standard curve from the relationship between Hoechst 33342 concentration and fluorescence intensity of U-937 cells (Figure 8(d)). The mean fluorescence intensity of the cells isolated from the peritoneal cavity 20 or 60 hrs after the administration was 4.78 or 47.61 per 10000 cells, respectively. The calculated concentration of Hoechst 33342 was 40.1 ng/mL after 20 hrs or 491.0 ng/mL after 60 hrs.

In the present study we have used Hoechst 33342-incorporated PLGA to identify, isolate, and characterize cells exposed to this fluorescent dye. The nuclear staining of Hoechst 33342 *in vivo* is a powerful marker for the isolation of cells from blood, ascites, pleural effusions, and even tissues when the tissue dissociation and cell isolation protocol is established. In addition, we can also collect cells that are negative for fluorescence. Once the various cells have been isolated, they can be analyzed for cell type and expression of specific molecules such as surface markers that may be important in cell targeting. One major limitation of the present approach is that Hoechst 33342 used as an imitating drug will be different from the actual drug in terms of molecular weight, structure, electrical charge, and/or presence/absence of specificity for a target molecule. Nonetheless, the present approach is useful for investigating the likely distribution of released materials from individual PLGA particles in the microenvironment of target tissues.

## 4. Conclusion

The present study successfully demonstrated that Hoechst 33342-incorporated PLGA particles can be used to simulate the drug exposure of cells *in situ*. We isolated cells exposed to this fluorescent dye as well as those that were not. These two classes of cells can then be further characterized, especially with regard to the expression of specific molecules that may be important in the targeting mechanism. The present approach may provide essential information concerning cell targeting in any type of PLGA DDS.

## Abbreviations

DDS: Drug delivery system
PLGA: Poly lactic-co-glycolic acids
D-MEM: Dulbecco's Modified Eagle Medium
EDTA: Ethylenediaminetetraacetic acid
FCS: Fetal calf serum
Dio: 3,3′-Dioctadecyloxacarbocyanine perchlorate
PBS: Phosphate-buffered saline.
